# Simple Smartphone-Based Assessment of Gait Characteristics in Parkinson Disease: Validation Study

**DOI:** 10.2196/25451

**Published:** 2021-02-19

**Authors:** Dongning Su, Zhu Liu, Xin Jiang, Fangzhao Zhang, Wanting Yu, Huizi Ma, Chunxue Wang, Zhan Wang, Xuemei Wang, Wanli Hu, Brad Manor, Tao Feng, Junhong Zhou

**Affiliations:** 1 Department of Neurology Beijing Tiantan Hospital Beijing China; 2 The Second Clinical Medical College Jinan University Guangzhou China; 3 Department of Geriatrics Shenzhen People’s Hospital Shenzhen, Guangdong China; 4 The First Affiliated Hospital Southern University of Science and Technology Shenzhen, Guangdong China; 5 Department of Computer Science The University of British Columbia Vancouver, BC Canada; 6 Hinda and Arthur Marcus Institute for Aging Research Hebrew SeniorLife Roslindale, MA United States; 7 Department of Hematology and Oncology Jingxi Campus, Capital Medical University Beijing ChaoYang Hospital Beijing China; 8 Beth Israel Deaconess Medical Center Boston, MA United States; 9 Harvard Medical School Boston, MA United States

**Keywords:** smartphone, gait, stride time (variability), validity, Parkinson disease

## Abstract

**Background:**

Parkinson disease (PD) is a common movement disorder. Patients with PD have multiple gait impairments that result in an increased risk of falls and diminished quality of life. Therefore, gait measurement is important for the management of PD.

**Objective:**

We previously developed a smartphone-based dual-task gait assessment that was validated in healthy adults. The aim of this study was to test the validity of this gait assessment in people with PD, and to examine the association between app-derived gait metrics and the clinical and functional characteristics of PD.

**Methods:**

Fifty-two participants with clinically diagnosed PD completed assessments of walking, Movement Disorder Society Unified Parkinson Disease Rating Scale III (UPDRS III), Montreal Cognitive Assessment (MoCA), Hamilton Anxiety (HAM-A), and Hamilton Depression (HAM-D) rating scale tests. Participants followed multimedia instructions provided by the app to complete two 20-meter trials each of walking normally (single task) and walking while performing a serial subtraction dual task (dual task). Gait data were simultaneously collected with the app and gold-standard wearable motion sensors. Stride times and stride time variability were derived from the acceleration and angular velocity signal acquired from the internal motion sensor of the phone and from the wearable sensor system.

**Results:**

High correlations were observed between the stride time and stride time variability derived from the app and from the gold-standard system (*r*=0.98-0.99, *P*<.001), revealing excellent validity of the app-based gait assessment in PD. Compared with those from the single-task condition, the stride time (*F_1,103_*=14.1, *P*<.001) and stride time variability (*F_1,103_*=6.8, *P*=.008) in the dual-task condition were significantly greater. Participants who walked with greater stride time variability exhibited a greater UPDRS III total score (single task: β=.39, *P*<.001; dual task: β=.37, *P*=.01), HAM-A (single-task: β=.49, *P*=.007; dual-task: β=.48, *P*=.009), and HAM-D (single task: β=.44, *P*=.01; dual task: β=.49, *P*=.009). Moreover, those with greater dual-task stride time variability (β=.48, *P*=.001) or dual-task cost of stride time variability (β=.44, *P*=.004) exhibited lower MoCA scores.

**Conclusions:**

A smartphone-based gait assessment can be used to provide meaningful metrics of single- and dual-task gait that are associated with disease severity and functional outcomes in individuals with PD.

## Introduction

Parkinson disease (PD) is a common neurodegenerative disease associated with numerous movement disorders and symptoms. One of the most significant disorders of PD is abnormal gait [1], which includes slowed walking speed, increased variability in stride timing (ie, stride time variability), and festination. These gait abnormalities have been linked to disease severity [2], and are on the causal pathway to an increased risk of falls [3], mortality, and morbidity [4]. As such, the functional status of patients suffering from PD, including gait, needs to be carefully assessed and considered for appropriate disease management.

Considerable effort has focused on the measurement of gait and mobility. Clinical rating scales [5,6] or stopwatch-based measurements (eg, timed-up-and-go test [7]) have been widely used to characterize gait in PD. However, these types of assessments are limited by subjective bias from clinicians, often have poor reliability, and are often insensitive to subtle pathological changes in PD. Recently, more advanced technologies have been developed to measure gait using specialized equipment such as wearable sensor systems or motion capture systems. This instrumented type of measurement can provide sophisticated and objective gait analysis with excellent reliability. For example, Mancini and colleagues [8] showed that using six wearable motion sensors consisting of a gyroscope, accelerometer, and digital compass attached on the left and right wrists, chest, lumber, and left and right shanks can accurately measure the temporal and spatial metrics of gait in multiple cohorts. However, such assessments are typically limited to clinical and laboratory settings, and require in-person contact with trained study personnel to reliably administer protocols and standardized instructions [9,10]. Moreover, such instrumented techniques do not afford regular gait assessments in large numbers of people due to the testing constraints, especially for those who are unable to utilize personal or public transportation and for those who live far from clinical centers or hospitals. Therefore, novel, easy-to-use, cost-effective, and scalable approaches for gait measurement in PD are needed.

With progress in smartphone technology, the inertial measurement unit (IMU) of smartphones—which consists of a 3D accelerometer, 3D gyroscope, and digital compass—has been utilized to capture movement associated with gait. Several studies have shown that the IMU of smartphones can accurately and reliably measure motion of the body in younger and older adults, as well as in those with PD [11-16]. For example, Ellis et al [15] showed that using the IMU of Apple iPod Touch secured on the navel can reliably and accurately measure gait in participants with and without PD. However, these approaches require the phone to be tightly secured to one part of the body and the studies were limited to assessments in laboratory environments with the need of trained study personnel to appropriately secure the phone using specialized equipment and to verbally provide standardized instructions to the participants.

Our team recently created a smartphone-based app enabling automatically guided assessment of gait when walking normally (ie, single task) and while performing a serial-subtraction cognitive task simultaneously (ie, dual task). The app was designed to provide standardized multimedia instructions to the user throughout the test to minimize the need for trained study personnel to administer the tests. Moreover, the assessment is completed with the phone placed in the user’s pants pocket, thereby removing the need for additional equipment to secure the phone to the body with a predetermined orientation. We previously demonstrated the validity and reliability of this app-based approach to measure gait characteristics in healthy adults [17]. The aim of this study was to determine the validity of using this app-based approach to gait assessment in people with PD, and to establish the association between app-derived gait metrics (eg, stride time, stride time variability) and the performance of several clinical characteristics in patients suffering from PD.

## Methods

### Participants

Fifty-two patients diagnosed with idiopathic PD by clinicians of the Department of Movement Disorders at Beijing Tiantan Hospital (Beijing, China) completed this study. All participants provided written informed consent as approved by the Beijing Tiantan Hospital Institutional Review Board. The inclusion criteria were: (1) aged between 25 and 80 years, (2) clinically diagnosed with PD using the 2015 Movement Disorder Society diagnosis criteria [1], and (3) the ability to walk for 1 minute without ambulatory or personal assistance. The exclusion criteria were: (1) presence of other overt neurological diseases such as dementia or stroke; (2) orthopedic impairments, history or presence of ulceration, amputation, or other painful symptoms in the lower extremities associated with impairment in gait; (3) self-reported diabetes mellitus or cardiovascular disease that may further alter gait; (4) drug or alcohol abuse; or (5) inability to understand the study procedure.

### Smartphone App

**Figure 1:**
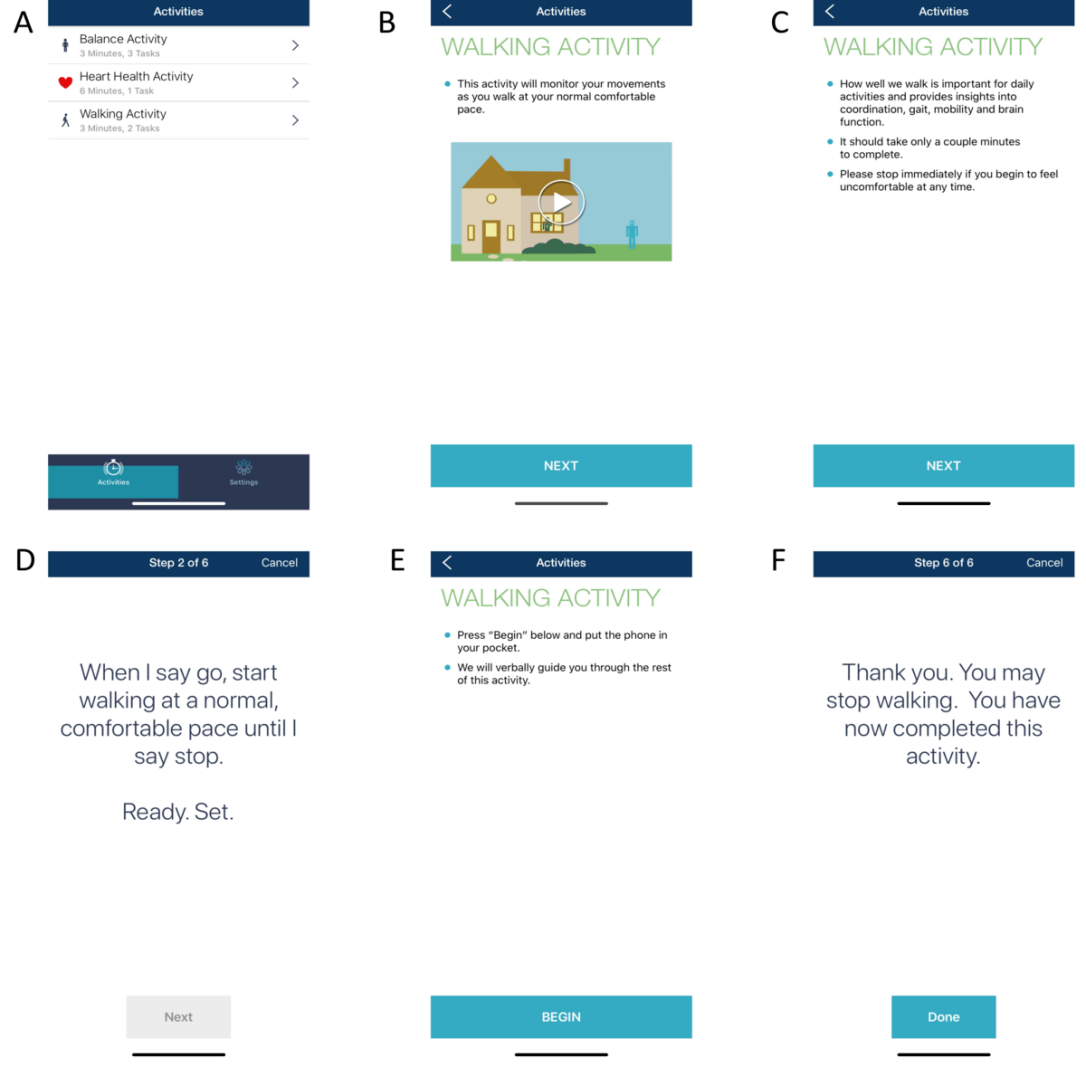
Screenshots of the smartphone gait and balance assessment app. The app, consisting of automatic animated, text, and voice instructions to users can measure the standing balance, 6-minute walking, and 45-second single- and dual-task walking performance (A). Before the walking test starts, users must first watch a comprehensive animation to introduce the testing procedure (B). Then, users must read several screens of text instruction (C). After reading the instruction, they press “begin” (D) and place the phone into the pocket of their pants to initiate the test. They then complete the walking trials following voice cues (including the message shown in panel E and the random 3-digit number provided in the dual-task condition). After pressing “Done” (F), the motion data of walking captured by the smartphone are automatically uploaded to a cloud-based server for storage and offline analysis.

The app was designed for use on the iPhone iOS platform. The goal of the app was to recreate standard laboratory-based assessments of standing and walking for use in the laboratory, clinics, and other nonlaboratory remote environments. Here, we focused on the functionality of the app to measure gait in participants with PD. The full description of the app, as well as the validity and reliability of the gait assessment in healthy adults, has been previously reported [17]. The previous validation study demonstrated that the app and customized-designed analytic approach can effectively quantify the stride time and stride time variability of gait during both single- and dual-task walking conditions as accurately as gold-standard laboratory instrumentation. The app-based assessment starts with an animated movie that provides a general overview of the assessment (developed by Wondros Inc, Los Angeles, CA), followed by several on-screen text step-by-step instructions. After watching the animation and reading the text instructions, participants press “Begin” and are instructed to place the phone into the pocket of their pants or shorts. The phone speaker then provides voice instructions and cues, guiding the participant through a 45-second trial of single-task walking and dual-task walking at their preferred speed (Figure 1). The dual-task walking involved asking the users to perform a verbalized serial subtraction of threes from a randomly generated 3-digit starting number when walking at their preferred speed. A 30-second rest between each trial was provided. Trial start and end “beep” cues triggered acquisition of the accelerometer, gyroscope, and compass data to the phone’s internal storage capacity. These data were automatically uploaded via Wi-Fi or a cellular service to a cloud-based data server immediately following the test for offline analyses [17] (Figure 1).

### Study Procedures

#### Setting and Design

All assessments were completed in the neurological clinics of Tiantan Hospital. All participants completed the tests in “medication-off” state, as defined by withholding their levodopa medication for at least 8 hours. The functional tests and clinical rating scales were completed before the walking assessment for all participants. Participants were given sufficient rest (at least 10 minutes) between each test to eliminate the effects of fatigue on test performance.

#### Walking Assessment

**Figure 2:**
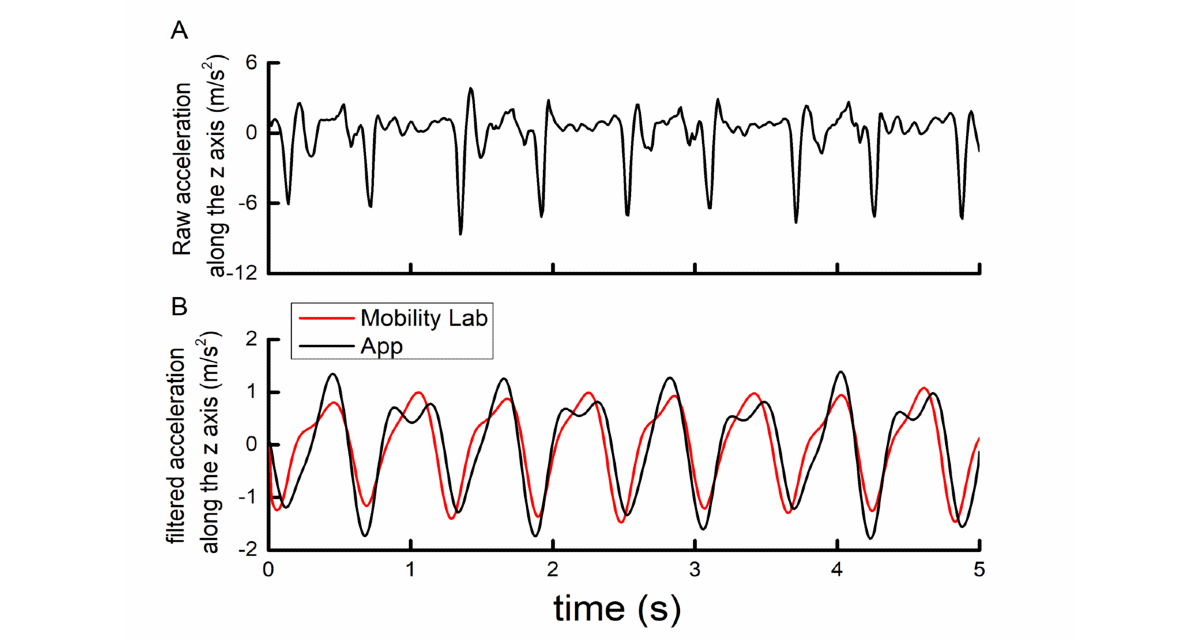
Example of raw (A) and filtered smartphone- (black) and Mobility Lab (red)–recorded acceleration signals (B) along the Earth coordinate system vertical axis during straight walking for 5 seconds.

The walking assessment was completed along a straight 10-meter hallway of the hospital. Participants were instructed to wear comfortable shoes and pants or shorts with pockets. Each participant completed the app assessment twice, with each assessment entailing one trial of single-task walking and one trial of dual-task walking at the participant’s preferred speed. During each trial, participants were asked to walk down the 10-meter hallway, turn 180 degrees, and walk back to the start*.* Therefore*,* the total length of straight walking in each trial was 20 meters. The trial order was randomized within each pair of trials. Participants utilized the app instructions to initiate and complete each trial. Study personnel oversaw the safety of participants without providing specific instructions. To assess the validity of gait metrics derived from the app, the Mobility Lab system (APDM, Seattle, WA), a widely used gold-standard and commercialized system of gait measurement, was also used to assess gait kinematics of each trial (Figure 2) [8]. This system consisted of three sensors: one secured over the lumbar back and two others secured to the top of each foot with Velcro straps.

#### Unified Parkinson Disease Rating Scale Part III

The Unified Parkinson Disease Rating Scale III (UPDRS III) was completed to assess PD severity. The UPDRS III assesses multiple aspects of function, including mental and mood function, motor control, activities of daily living, and complications of therapy. The total score of UPDRS III, ranging from 0 to 28, was used in the analysis, with greater scores reflecting more severe PD.

#### Montreal Cognitive Assessment

The Montreal Cognitive Assessment (MoCA) was used to examine global cognitive function, including visuospatial, executive function, attention/working memory, episodic memory, and language. The MoCA total score, which ranges from 0 to 30, was used for analysis, with lower scores reflecting worse cognitive function.

#### Hamilton Anxiety and Depression Rating Scales

Participants completed the Hamilton Anxiety (HAM-A) [18] and Hamilton Depression (HAM-D) [19] scales to assess their mood. HAM-A consists of 14 items assessing symptoms related to anxiety and HAM-D consists of 21 items (17 of them used in this study) assessing symptoms related to depression. Each item of HAM-A was scored between 0 (ie, not present) and 4 (severe), with a total range of 0 to 56. Nine items in HAM-D were scored between 0 and 4 and the other eight were scored between 0 and 2, with a range between 0 and 52. The total scores of HAM-A and HAM-D were used in the subsequent analysis, with greater scores reflecting more severe anxiety or depression.

#### Analysis of Gait Metrics

The pipeline of signal processing and data analysis was described in our previous paper [17]. Briefly, kinematic data related to gait (ie, the acceleration and angular velocity time series) were captured by the accelerometer and gyroscope within the IMU of the smartphone and from the IMUs within the Mobility Lab sensors at a sampling frequency of 100 Hz. The raw 3D acceleration and angular velocity time series captured by the app were then each transformed from the device coordinate to an Earth-based coordinate system using the quaternion rotation matrix [20]. By doing so, the *Z*-axis was thus approximately vertical to the ground, regardless of the orientation of the phone placed in the pocket (Figure 2A). Each rotated time series was then filtered using a low-pass Butterworth filter with a cut-off frequency of 3 Hz. These filtered time series, containing peaks that oscillated with different amplitudes, aligned with the time series recorded by the Mobility Lab system very well (Figure 2B). Turns of 180 degrees were automatically detected and removed from the analysis as described previously [17], so that gait was only analyzed during periods of straight-line walking. We then identified each heel strike and toe-off of the phone-side leg, which we previously determined to correspond with the trough nadir following each relatively high peak and the trough nadir following each relatively low peak [17].

Finally, stride time and stride time variability were calculated for each trial. Stride time is typically defined as the time between two consecutive heel strikes of one foot, and thus relates to one complete gait cycle. Stride time variability is defined using the ratio of the SD of the stride times to the mean stride time within each walking trial. Longer stride time and greater stride time variability within a given walking condition (ie, single or dual task) have each been associated with aging [21], incidence of movement disorders (including PD) [22], the development of falls [3], and even the likelihood of future cognitive decline [23]. We also calculated the dual-task “costs” to stride time and stride time variability as the percent change of each metric from the single- to dual-task walking condition. A greater dual-task cost reflected a greater dual-task decrement in walking performance. The averaged stride time, stride time variability, and dual-task cost across two trials for each participant were then used in the following analyses.

Similar gait metrics were derived from the Mobility Lab data using the software platform provided with the system.

### Statistical Analysis

Statistical analyses were performed using JMP Pro 14 software (SAS Institute, Cary, NC). The significance level was set to *P*<.05 for all analyses.

We examined the validity of app-derived stride time and stride time variability by assessing the correlation between app-derived metrics and the corresponding metrics derived from the Mobility Lab system using Pearson correlation analysis. The validity in single- and dual-task walking conditions was examined in separate models. We also calculated the absolute difference between the metrics measured by the app and by Mobility Lab. In addition, we examined the effects of task condition on stride time and stride time variability in separate two-way analysis of variance (ANOVA) models. The model factor was the task condition (ie, single and dual task), and the dependent variables were the stride time and stride time variability in each model, respectively. Each of these ANOVA models was adjusted for age, sex, and years of formal education.

Linear regression models were then used to examine the relationships between gait metrics measured by the app and scores of the UPDRS III, MoCA, HAM-A, and HAM-D scales. Age, sex, and years of formal education were included as covariates in each model.

## Results

### Participant Characteristics

Participants ranged in age from 40 to 83 years. No adverse events or safety issues were reported during the study. Each participant successfully completed the walking trials following the instructions provided by the app with no difficulty. Table 1 shows the demographic, clinical, and functional characteristics, and app-measured gait metrics of participants. Compared to the single-task condition, the stride time (*F_1,103_*=14.1, *P*<.001) and stride time variability (*F_1,103_*=6.8, *P*=.008) of gait in the dual-task condition were significantly larger, indicating significant “interference” from the concurrent serial subtraction task on locomotor control. Each of these results was independent of covariance associated with age, sex, and education (*F_1,103_*<1.3, *P*>.66).

**Table 1:** Participants characteristics (N=52).

Demographics and gait metrics			Value
Female, n (%)		19 (37)	
Age (years), mean (SD)		63 (10)	
Education (years), mean (SD)		11 (3)	
Height (m), mean (SD)		1.7 (0.9)	
Body mass (kg), mean (SD)		70 (21)	
Duration of Parkinson disease (years), mean (SD)		6.4 (3.8)	
UPDRS III^a^		38.3 (15.1)	
MoCA^b^, mean (SD)		22 (4)	
HAM-A^c^, mean (SD)		11.8 (4)	
HAM-D^d^, mean (SD)		11.1 (5.1)	
**Stride time, mean (SD)**			
	single task (s)	1.09 (0.08)	
	dual task (s)	1.17 (0.12)	
	DTC^e^ (%)	2.7 (6)	
**Stride time variability^f^ , mean (SD)**			
	single task (%)	40 (22)	
	dual task (%)	63 (19)	
	DTC (%)	5.1 (39.4)	

^a^UPDRS III: Unified Parkinson Disease Rating Scale III.

^b^MoCA: Montreal Cognitive Assessment.

^c^HAM-A: Hamilton Anxiety Scale.

^d^HAM-D: Hamilton Depression Scale.

^e^DTC: dual task cost.

^f^Ratio of the SD to the mean of stride time.

### Validity and Variability of App-Derived Stride Time

**Figure 3:**
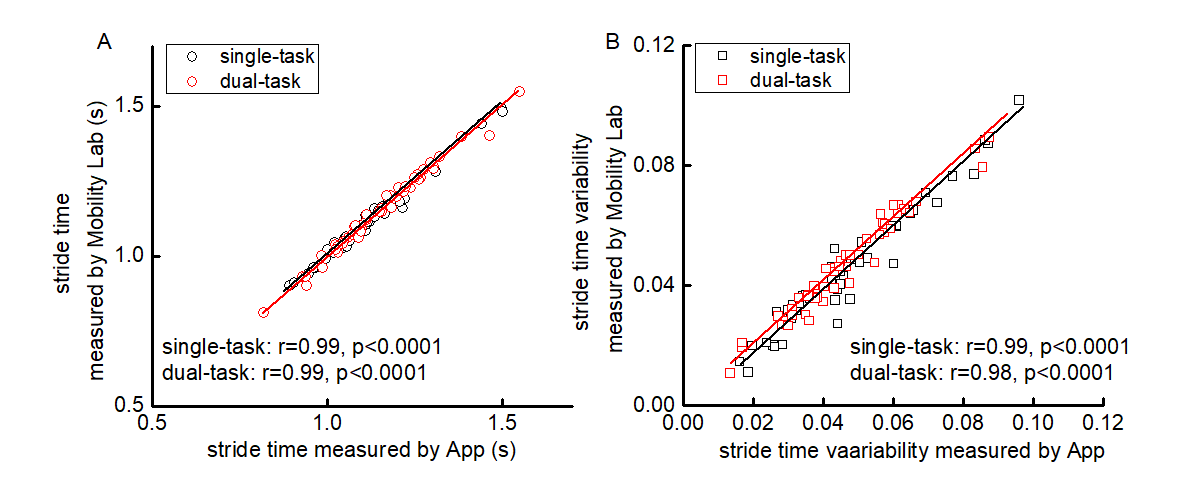
Correlation between stride time (A) and stride time variability (B) as measured by the app (x-axis) and the Mobility Lab system (y-axis) for each participant.

The average number of identified strides during the 20-meter straight-walking trials was 9.5 (SD 1.5). The absolute difference between the app and Mobility Lab measures was quite small (Table 2). Pearson correlation analysis revealed that app-derived stride time was strongly correlated with that derived from Mobility Lab under both the single-task (*r*=0.99, *P*<.001) and dual-task (*r*=0.99, *P*<.001) conditions (Figure 3A). Moreover, the app-derived stride time variability was strongly correlated with that derived from Mobility Lab in both single-task (*r*=0.99, *P*<.001) and dual-task (*r*=0.98, *P*<.001) conditions (Figure 3B). Taken together, the stride time and stride time variability derived from the app demonstrated excellent validity as compared with those derived from Mobility Lab for gait assessment in people with PD.

**Table 2:** Absolute difference between gait metrics derived from the app and Mobility Lab data.

Condition	Stride time (seconds), mean (SD)	Stride time variation (seconds), mean (SD)
Single task	0.01 (0.008)	0.003 (0.003)
Dual task	0.01 (0.009)	0.002 (0.002)

### Relationship of App-Derived Gait Metrics With Clinical and Functional Status

#### UPDRS-III

**Figure 4:**
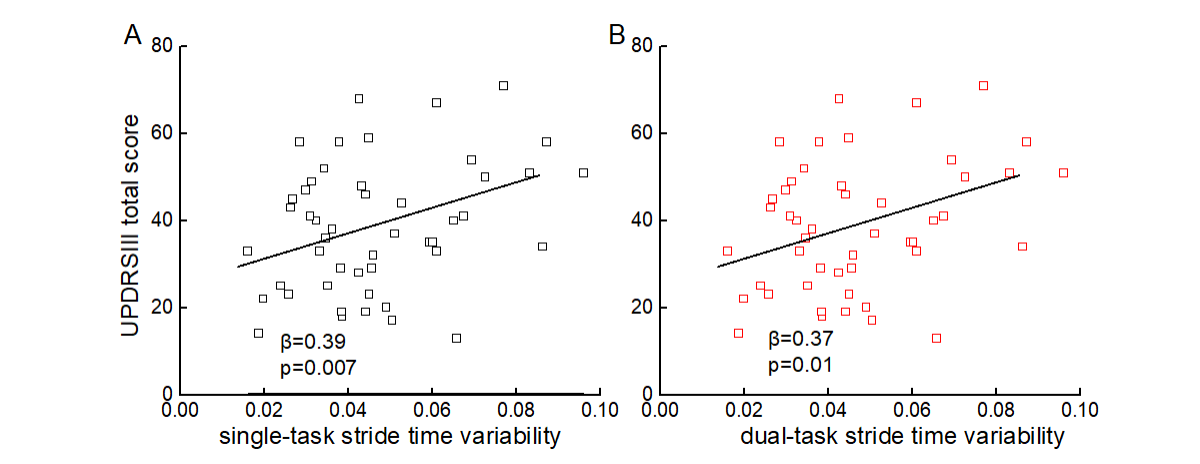
Association between single- (A) and dual-task (B) stride time variability and total score of Unified Parkinson Disease Rating Scale III (UPDRS III).

Linear regression models adjusted for age, sex, and duration of formal education demonstrated that the stride time variability of both single-task (β=.39, *P*<.001) and dual-task (β=.37, *P*=.01) walking was significantly correlated with the UPDRS-III total score (Figure 4). Participants with greater stride time variability tended to exhibit more severe PD as measured by the UPDRS III. No significant correlations were observed between other gait metrics (stride time in either condition, dual-task cost to stride time or stride time variability) and the UPDRS-III score (β=.12 to.21, *P*=.18 to .34).

#### MoCA

Linear regression models revealed that the stride time variability of dual-task walking (β=.48, *P*=.001) and the dual-task cost to stride time variability (β=.44, *P*=.004) were each correlated with the MoCA score. Participants with greater stride time variability in dual-task walking or greater dual-task cost had lower MoCA scores (ie, worse cognitive function). No such association was observed between other gait metrics and this measure of global cognitive function (β=.06 to .12, *P*=.42 to .53).

#### HAM-A and HAM-D

**Figure 5:**
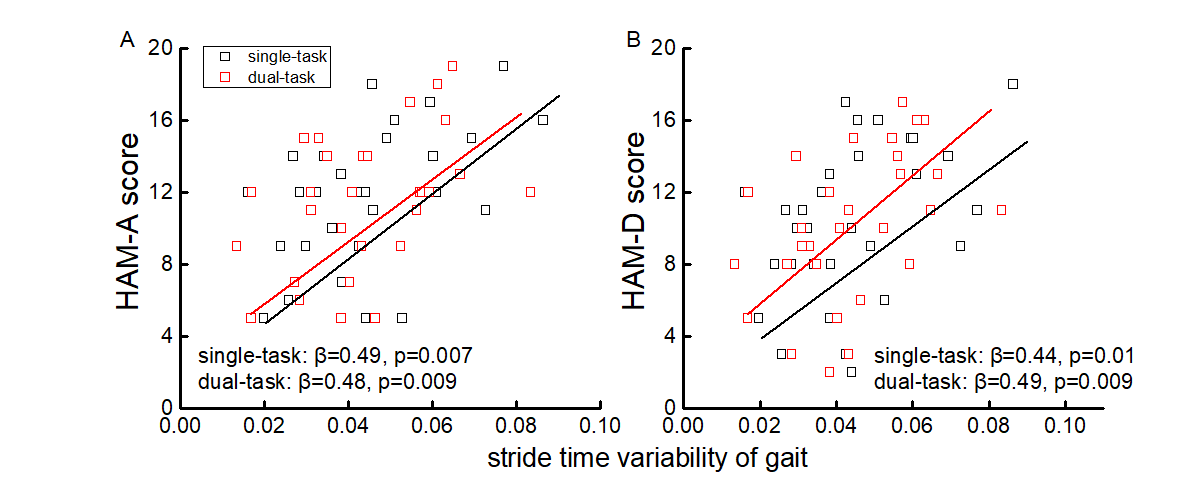
Association between single- and dual- task stride time variability and the score of the Hamilton Anxiety (HAM-A) (A) and Hamilton Depression (HAM-D) (B) scales. Greater scores reflect a worse mood status.

Stride time variability of both single- and dual-task walking was significantly correlated with HAM-A (single task: β=.49, *P*=.007; dual task: β=.48, *P*=.009) and HAM-D (single task: β=.44, *P*=.01; dual task: β=.49, *P*=.009). In each case, participants with greater stride time variability reported worse anxiety and more severe depressive symptoms (Figure 5).

## Discussion

This study provides a proof of concept that patients with PD can use a smartphone app by themselves to accurately assess gait during both single-task and cognitive dual-task walking conditions, with the phone placed in the front pocket of the pants or shorts. Moreover, gait metrics derived from app data, particularly under the dual-task condition, were associated with several important functional and clinical rating scales in PD. Such information may thus be used to help assess PD severity, its functional impact, and potentially the effectiveness of medication or other clinical interventions within this vulnerable population.

Typically, gait assessments are performed with low frequency (ie, only during in-person clinical visits, or only before and after a study intervention). However, recent studies have demonstrated that even within an individual, the characteristics of walking vary considerably within and between days. Such variance in function has been associated with multiple important outcomes [24-28]. For example, Leach et al [27] demonstrated that older adults with greater day-to-day variance in standing balance had lower cognitive function. Albrecht et al [28] reported that a single measurement of walking performance may cause misinterpretation of functional status in patients with multiple sclerosis due to the high day-to-day variance in gait. It is thus important to characterize gait with sufficiently high frequency. In-person assessments that require specialized equipment and trained personnel to administer tests do not lend themselves well to high-frequency monitoring [29-32]. The smartphone app-based gait measurement used in this study does not require specialized equipment beyond a smartphone and does not need trained personnel to administer the test. It also automatically uploads and stores acquired data via Wi-Fi or a cellular service, and is thus highly portable. Taken together, the app may therefore serve as an easy-to-use, widely accessible, and cost-effective tool for high-frequency assessment of gait within both laboratory and nonlaboratory settings.

Walking in everyday life often requires simultaneous performance of additional cognitive tasks such as speaking to others, thinking of questions, or reading signs in the environment. This dual tasking disrupts the performance of one or both tasks. Consistent with previous studies [17,33], we observed that in people with PD, gait is more unstable (ie, greater stride time variability) when dual tasking as compared to when walking quietly, revealing that the performance of the concurrent cognitive serial-subtraction task alters locomotor control. Mounting evidence has shown that walking performance in the dual-task condition can be used to characterize an individual’s capacity of cognitive-motor control, and can predict both fall risk and the incidence of dementia in older adults [34,35]. Consistent with these results, we observed that the stride time variability of gait in the dual-task condition and the dual-task cost to this metric were cross-sectionally associated with the severity of PD as assessed by the total score of UPDRS-III, the general cognitive function as measured by the MoCA score, and mood problems (ie, anxiety and depression as assessed by the HAM-A and HAM-D rating scales, respectively) in this cohort. Recent research efforts have provided evidence that gait and other movement disorders in PD, including freezing of gait (FOG), are not only motor issues, but arise in part because of deficits in cognitive functioning [36-40]. For example, Amboni and colleagues [39] observed that compared to those without FOG, participants with PD and FOG had significantly poorer performance of cognitive tasks, including frontal assessment battery, verbal fluency, and the 10-point clock test [39]. Therefore, the assessment of dual-task gait in PD promises to help the characterization of cognitive-motor functioning as it relates to gait and mobility in this population.

This study demonstrated the feasibility of using a smartphone app for gait assessment in PD, as well as the validity of app-derived gait metrics within this population. Several participants in this study had mild-to-moderate cognitive impairment as assessed by the MoCA (the mean score was 22) and all of the participants successfully completed the test without any reported difficulties. Future development and testing is nevertheless needed to optimize the design of the app for use in patients with PD that have more severe cognitive impairment, as well as for those with other comorbidities (eg, diabetes mellitus, cardiovascular disease, chronic pain), and those with limited vision, hearing loss, tremor, or other issues that may hinder the ability to interact with the smartphone. In this study, the association between gait metrics and functional characteristics was assessed cross-sectionally, and gait metrics were only assessed in person within the clinical setting. Future work is thus warranted to establish the usefulness of regular, smartphone-based gait assessments captured from remote settings (ie, patient homes). Work focused on remote assessment is of particular importance during the current COVID-19 pandemic as it promises to help maintain quality of care while reducing the spread of infectious disease. The usefulness of the app is also likely to be further optimized by validating other clinically meaningful metrics of gait (eg, gait speed and the asymmetry of gait); characterizing gait during turning [41]; detecting FOG, festination, or other movement abnormalities that may occur during walking; enabling the passive monitoring of gait throughout the day; and implementing other types of cognitive tasks (eg, auditory wording task [42]) into the dual-task walking paradigm.

## Abbreviations

ANOVA: analysis of variance
FOG: freezing of gait
HAM-A: Hamilton Anxiety Scale
HAM-D: Hamilton Depression Scale
IMU: inertial measurement unit
MoCA: Montreal Cognitive Assessment
PD: Parkinson disease
UPDRS III: Unified Parkinson Disease Rating Scale III
